# The Rapid Discrimination and Quality Assessment of Three *Zanthoxylum* Species Using ^1^H NMR Spectrometry

**DOI:** 10.1155/2020/3830258

**Published:** 2020-08-07

**Authors:** Hyeon Seok Jang, Birang Jeong, Seong Yeon Choi, Jiho Lee, Yong Soo Kwon, Heejung Yang

**Affiliations:** College of Pharmacy, Kangwon National University, Chuncheon 24341, Republic of Korea

## Abstract

The husks and fruits of *Zanthoxylum* species (Rutaceae) are the popular pungent and spicy ingredients of foods and the traditional medicines in many countries. Three *Zanthoxylum* species, *Z*. *bungeanum*, *Z*. *schinifolium*, and *Z*. *piperitum*, are distributed and intermixed with each other as “Zanthoxyli Pericarpium” in Korean markets. In the present study, we analyzed the ethyl acetate-soluble and nonpolar fractions of *Zanthoxylum* samples by ^1^H NMR spectrometry and performed a multivariate analysis for finding the discriminant markers between three species. Xanthoxylin was identified as the metabolic marker for the discrimination of *Zanthoxylum* species and quantified by the qNMR approach.

## 1. Introduction

*Zanthoxylum* species, Rutaceae, are the popular edible ingredients in Asian countries and are used as spices for spicy cuisine and medicinal herbs for treating many syndromes. In Korea, three *Zanthoxylum* species, *Z*. *bungeanum*, *Z*. *schinifolium*, and *Z*. *piperitum*, were distributed as “Zanthoxyli Pericarpium,” which are used as pungent condiments, spicy seasoning of foods, and medicinal herbs for stimulating intestinal motility and treating dyspepsia [1]. *Zanthoxylum* species contain bioactive alkaloids, aromatic and aliphatic amides, and phenolic compounds with anti-inflammation, antiplatelet aggregation, antioxidant, and antitumor activities [2, 3]. However, three *Zanthoxylum* species have different chemical profiles affecting the quality of biological activities and characteristic flavours and hence should be separately used [4].

Nuclear magnetic resonance (NMR) spectroscopy is a versatile technique in metabolomics and can be applied to monitor the metabolic profiles and quantify the target molecules in the biofluid matrix, as well as to elucidate the structures of small and large molecules in natural products chemistry. NMR spectroscopy yields highly accurate, precise, and reproducible spectroscopic data compared with other spectroscopic techniques, mass spectrometry [5]. Recently, quantitative NMR (qNMR) is utilized to determine the concentration and purity of one or more metabolites in the sample and applied to check the quality control of natural products [6]. The qNMR method is a rapid analysis and nondestructive with easy sample preparation which enables the simultaneous quantification of multiple metabolites in the sample without the calibration curves of reference standards [7].

In the present study, we performed the multivariate analysis (MVA) to simultaneously discriminate three *Zanthoxylum* species which were expected to have different NMR profiles due to their own biosynthetic systems and tried to discover and quantitate the metabolic marker which was contributed to the discrimination of three species using by the qNMR approach.

## 2. Materials and Methods

### 2.1. Plant Materials

Thirty-five dried *Zanthoxylum* samples, 13 of *Z*. *bungeanum*, 6 of *Z*. *schinifolium*, and 16 of *Z*. *piperitum*, were collected from March to September 2016, being purchased from Korea traditional herb markets (Kyungdong market, Seoul, Korea). *Z. piperitum* and *Z. schinifolium* samples were originated from Korea and *Z. bungeanum* from China. They were identified by Professor Yong Soo Kwon, College of Pharmacy of Kangwon National University, and deposited in the Herbarium of the College of Pharmacy of Kangwon National University (Supplementary Materials Table S1). One gram of the samples was extracted with 10 ml of 100% MeOH in an ultrasonic bath for 3 hours, freeze-dried, and kept at −80°C to ensure their sample integrity for further analysis.

### 2.2. NMR Analysis

10 mg of freeze-dried samples and 1 mg of the reference standard, xanthoxylin (Corescience Inc., Seoul, Korea), were dissolved in 1 ml of CDCl_3_ (euriso-top, Saint-Aubin, French) mixed with the internal standard, methyl 3,5-dinitrobenzoate (1⟶20) (Alfa Aesar, Massachustts, USA). All the NMR experiments were performed with a Bruker Avance II 600 spectrometer (Bruker Biospin, Rheinstetten, Germany) equipped with a 5 mm BBO probe with *Z* gradient in the Central Laboratory of Kangwon National University. NMR spectra were acquired at 298 K. Data acquisition and processing were performed with Bruker Topspin 3.0. ^1^H NMR spectra were acquired using the Bruker zg30 pulse program with the following settings: relaxation delay (d1), 1 s; flip angle, 30°; acquisition time, 1.33 s; free induction decay (FID) data points, 64 kbytes; spectral width, 20.55 ppm; and number of scans, 256. In all cases, the acquired FIDs were Fourier transformed to yield spectra with 128 kbyte data points (zero filling). Manual phase correction and baseline correction were always used. Chemical shift values were referenced to the calibration standard (TMS) signal.

### 2.3. Multivariate Analysis

^1^H NMR spectra were processed by binning 0.001 ppm width and normalized by the integration of the peaks separated at *δ* 9.16∼9.25 ppm of the internal standard, methyl 3,5-dinitrobenzoate, by Mnova (ver. 6.0.2, Mestrelab Research, Compostela, Spain) software. The processed NMR spectra were bucketed with more than 3.00 ppm, excluding the residual MeOH at 3.45∼3.51 ppm, methyl 3,5-dinitrobenzoate at 4.05∼4.10 and 9.16∼9.25 ppm, and the residual non-deuterated chloroform signals at 7.24∼7.28 ppm, respectively. The multivariate analyses, PCA and PLS-DA, were performed with *R* package ropls (ver. 1.14.0). The variables of the processed data matrix were scaled to the Pareto scaling method. In PLS-DA model, the R2X, R2Y, and Q2 values were calculated to describe the total variation in *X* and the variation in the response variable Y, and the predictive ability of the models, respectively. The score and loading plots were visualized in the optimal three-dimensional space for identifying differences among groups by *R* package rgl (ver. 0.99.16).

### 2.4. Quantitative NMR (qNMR) Analysis of Xanthoxylin

The qNMR analysis for xanthoxylin was performed with a slight modification to a previously published analysis [8]. We used methyl 3,5-dinitrobenzoate (1⟶20) as the internal standard for absolute quantification. For identifying the purest signal and non-overlapped signals of xanthoxylin in the standard and *Zanthoxylum* samples, the purities of all the signals of xanthoxylin were determined using the following equation:

(1) $$\begin{array}{c} P_{F}\;\left[\%\right]\;=\;\frac{n_{\text{IS}}\cdot {\text{Int}}_{F}\cdot {\text{MW}}_{F}\cdot m_{\text{IS}}}{n_{F}\cdot {\text{Int}}_{\text{IS}}\cdot {\text{MW}}_{\text{IS}}\cdot m_{F}}\;\cdot \;P_{\text{IS}}, \end{array}$$

where Int is the integral, MW is the molecular weight, *m* is the mass, *n* is the number of protons, *P* is the purity expressed as %, IS is the internal standard, and F is xanthoxylin. The intraday and interday variabilities of xanthoxylin were examined by three replicate experiments at a concentration of 10 mg/ml in CDCl3 mixed with the internal standard, methyl 3,5-dinitrobenzoate (1⟶20) in one day for the intraday test and three consecutive days (1, 3, and 5 days) for the interday test. The relative standard deviations (RSD) were calculated as a measure of precision.

## 3. Results and Discussion

**Figure 1:**
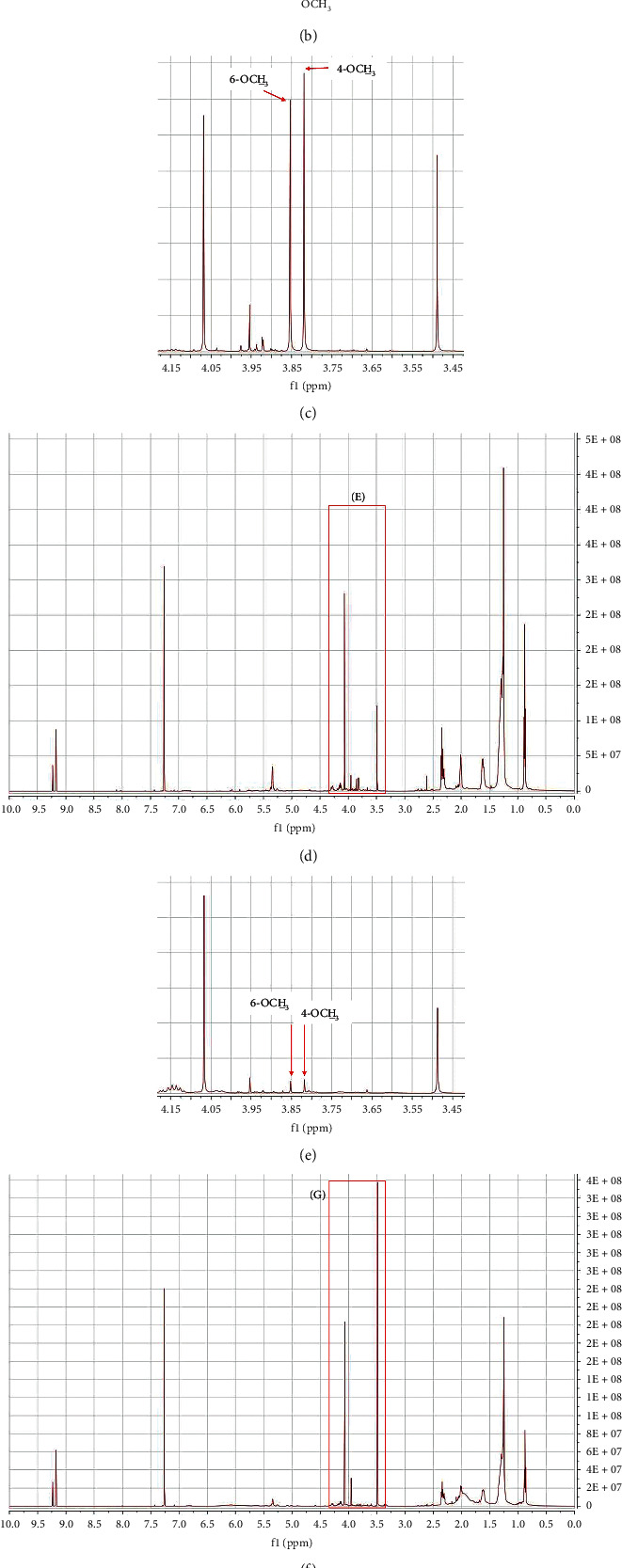
^1^H NMR spectra of three *Zanthoxylum* species—*Z*. *bungeanum* (a, c), *Z*. *schinifolium* (d, e), and *Z*. *piperitum* (f, g)—and the structure of xanthoxylin (b). The ^1^H NMR spectra corresponding to the signals of 4-OCH_3_ and 6-OCH_3_ in (a), (d), and (f) were magnified to (c), (e), and (g), respectively.

The NMR experimental parameters were optimized to obtain clear resolution and separation of peaks (see Section 2). Methyl 3,5-dinitrobenzoate was added in NMR solvent as the internal standard of which the chemical shifts at *δ* 9.2 (2H, *d*, *J* = 2.13 Hz) and 9.3 (^1^H, *t*, *J* = 2.13 Hz) ppm were totally not overlapped with other peaks (Figure 1). In the ^1^H NMR of *Zanthoxylum* samples, we excluded the up-fielded peaks in the range of *δ* 0.8∼2.5 ppm which were tentatively expected as the aliphatic lipids and overwhelmed other minor peaks. Therefore, we processed the down-fielded peaks in the ranges of above 3.0 ppm which were normalized by the integral of two peaks at *δ* 9.2 (2H, *d*, *J* = 2.13 Hz) and 9.3 (^1^H, *t*, *J* = 2.13 Hz) of the internal standard for the further study.

**Figure 2:**
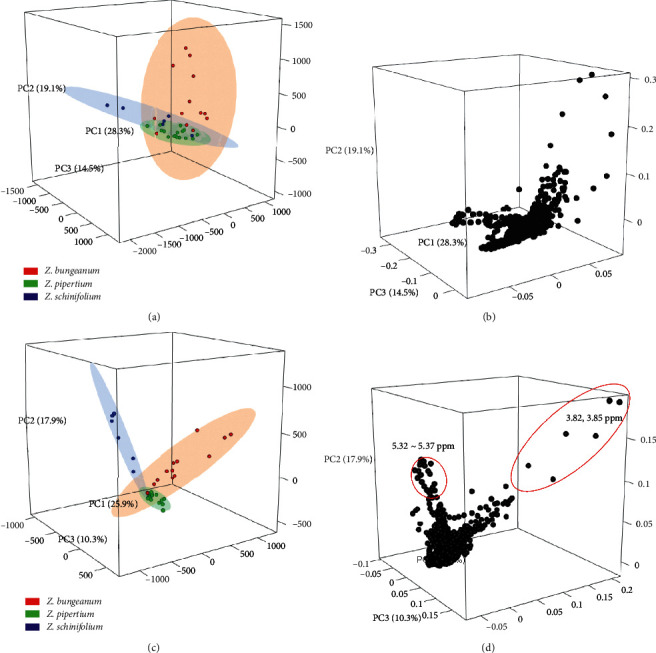
The multivariate plots of three *Zanthoxylum* species based on the ^1^H NMR spectra. (a) The PCA score plot with 95% density ellipses. (b) The PCA loading plot. (c) The PLS-DA score plot with 95% density ellipses. (d) The PLS-DA loading plot. The red circles in the PLS-DA loading plot show the variables contributing to the separation of *Z*. *bungeanum* and *Z*. *schinifolium* samples.

After the processing steps, such as peaks alignment, normalization, and binning, of the NMR spectral data, we excluded the solvent signals at *δ* 3.45∼3.51 ppm of the residual MeOH, *δ* 4.05∼4.10 and *δ* 9.16∼9.25 ppm of methyl 3,5-dinitrobenzoate, and *δ* 7.24∼7.28 ppm of the residual non-deuterated chloroform which hindered the interpretation of minor peaks in samples. Firstly, the data matrix was subjected to the unsupervised multivariate analysis (MVA) and the explorative principal components analysis (PCA) to investigate the general trend of clustering between three species, but the samples labelled with species were not clearly clustered by major principal component (PC) 1 (Figure 2). Though *Z*. *bungeanum* samples were roughly separated from two species along PC2, the scores for *Z*. *piperitum* and *Z*. *schinifolium* were nearly identical. *Z*. *piperitum* samples were clustered closer together than the other two species, and their qualities were more consistent than others. In the loading plot, *δ* 3.82 and 3.85 ppm (PC1 > 0.15 and PC2 > 0.11), 4.11 (PC1 > 0.06 and PC2 < -0.03), and 5.34 ppm (PC1 < −0.06 and PC2 > 0.11) were contributed as the significant variables. The supervised partial least squares discriminant analysis (PLS-DA) (R2X = 0.54; R2Y = 0.71; *Q*2 = 0.61) improved the separation between species (Figure 2). In consistent with PCA results, *Z*. *piperitum* samples were more compact than *Z*. *bungeanum* and *Z*. *schinifolium*, along the three major predictive components. The groups corresponding to *Z*. *schinifolium* and *Z*. *piperitum* samples were observed at negative values of PC1, while *Z*. *bungeanum* samples were found at positive ones of PC1. Next, we investigated the loading plot for identifying the metabolic markers contributing to the separation of three species in the score plot in PLS-DA. The variables for *δ* 3.82 and 3.85 ppm (PC1 > 0.1, PC2 > 0.1, and PC3 > 0.12) and for *δ* 5.34 ppm (PC1 < −0.05, PC2 > 0.11 and PC3 > 0.02) were significantly scattered in the direction of *Z*. *bungeanum* and *Z*. *schinifolium*, respectively, which were similar to the PCA results.

The signals at *δ* 5.32∼5.37 ppm were tentatively predicted as the protons attached to the olefinic carbons, but the signals at *δ* 3.82 and 3.85 ppm were identified as a simple phenolic compound, xanthoxylin, which was confirmed with the ^1^H NMR spectrum of the standard (see Figure S1 in Supplementary Materials) [9–12]. Precision tests of the two signals in xanthoxylin were performed (Table 1). The intraday and interday variations were less than 4.43 and 2.55, respectively. The signals of two methoxy protons were sufficient to use as a quantitative standard in *Zanthoxylum* species. Xanthoxylin in three *Zanthoxylum* species was calculated by the qNMR method. Although the variance was large within the same species, xanthoxylin was significantly more abundant in *Z*. *bungeanum* than the other two species (Table 2).

## 4. Conclusions

qNMR is the versatile spectroscopic technique to quantify the target molecule in the mixture of metabolites in the extract and to simultaneously inspect the quantitative changes of metabolites with the multivariate analysis. This method can be applied to analyze huge metabolites in natural products samples. In the present study, we measured the 1H NMR spectra of 35 samples of three *Zanthoxlyum* species and developed the rapid discrimination method of three *Zanthoxlyum* species by ^1^H NMR spectrometry. As a result, the metabolic marker, xanthoxylin, contributed to the discrimination of three species. This simple approach could be applied to the quality assessment of dried *Zanthoxlyum* samples and their extract-containing products.

## Figures and Tables

**Table 1:** Precision for ^1^H NMR signals of xanthoxylin for proton (ppm); intraday, RSD (%); and interday, RSD (%).

Proton (ppm)	Intraday, RSD (%)	Interday, RSD (%)
4-OCH_3_ (3.82)	4.43	2.50
6-OCH_3_ (3.85)	4.42	2.55

**Table 2:** The content of xanthoxylin for 16 elutions in three *Zanthoxylum* species: *Z. piperitum*, *Z. schinifolium*, and *Z. bungeanum*.

No.	*Z. piperitum*	*Z. schinifolium*	*Z. bungeanum*
	(mg/ml)		
1	0.03	0.14	0.05
2	0.03	0.13	0.08
3	0.03	0.10	0.58
4	0.06	0.12	0.37
5	0.04	0.03	1.13
6	0.02	0.09	1.46
7	0.02		1.13
8	0.04		0.40
9	0.06		1.43
10	0.08		0.47
11	0.06		0.35
12	0.07		0.11
13	0.03		0.06
14	0.02		
15	0.05		
16	0.05		
Mean	0.04	0.10	0.59
SD	0.02	0.04	0.52

## Data Availability

The data used to support the findings of this study are available from the corresponding author upon request.

## Supplementary Materials

Supplementary Materials Table S1: the herbarium records of *Zanthoxylum* species. Figure S1: the ^1^H NMR spectrum of xanthoxylin mixed with the internal standard, methyl 3,5-dinitrobenzoate.
