# Supplementary material for: The Rapid Discrimination and Quality Assessment of Three Zanthoxylum Species Using 1H NMR Spectrometry
Source: Int J Anal Chem. 2020 Aug 7;2020:3830258. doi: 10.1155/2020/3830258 (PMC7428930; doi:10.1155/2020/3830258)

Table S1 : The herbarium record of *Zanthoxylum* species

Herbarium Record	Species	Origin	Herbarium Record	Species	Origin	Herbarium Record	Species	Origin
KNUPH-ZP-1	*Z. piperitum*	Korea	KNUPH-ZB-1	*Z. bungeanum*	China	KNUPH-ZS-1	*Z. schinifolium*	Korea
KNUPH-ZP-2	*Z. piperitum*	Korea	KNUPH-ZB-2	*Z. bungeanum*	China	KNUPH-ZS-2	*Z. schinifolium*	Korea
KNUPH-ZP-3	*Z. piperitum*	Korea	KNUPH-ZB-3	*Z. bungeanum*	China	KNUPH-ZS-3	*Z. schinifolium*	Korea
KNUPH-ZP-4	*Z. piperitum*	Korea	KNUPH-ZB-4	*Z. bungeanum*	China	KNUPH-ZS-4	*Z. schinifolium*	Korea
KNUPH-ZP-5	*Z. piperitum*	Korea	KNUPH-ZB-5	*Z. bungeanum*	China	KNUPH-ZS-5	*Z. schinifolium*	Korea
KNUPH-ZP-6	*Z. piperitum*	Korea	KNUPH-ZB-6	*Z. bungeanum*	China	KNUPH-ZS-6	*Z. schinifolium*	Korea
KNUPH-ZP-7	*Z. piperitum*	Korea	KNUPH-ZB-7	*Z. bungeanum*	China			
KNUPH-ZP-8	*Z. piperitum*	Korea	KNUPH-ZB-8	*Z. bungeanum*	China			
KNUPH-ZP-9	*Z. piperitum*	Korea	KNUPH-ZB-9	*Z. bungeanum*	China			
KNUPH-ZP-10	*Z. piperitum*	Korea	KNUPH-ZB-10	*Z. bungeanum*	China			
KNUPH-ZP-11	*Z. piperitum*	Korea	KNUPH-ZB-11	*Z. bungeanum*	China			
KNUPH-ZP-12	*Z. piperitum*	Korea	KNUPH-ZB-12	*Z. bungeanum*	China			
KNUPH-ZP-13	*Z. piperitum*	Korea	KNUPH-ZB-13	*Z. bungeanum*	China			
KNUPH-ZP-14	*Z. piperitum*	Korea						
KNUPH-ZP-15	*Z. piperitum*	Korea						
KNUPH-ZP-16	*Z. piperitum*	Korea						

Figure S1: The ¹H NMR spectrum of xanthoxylin.

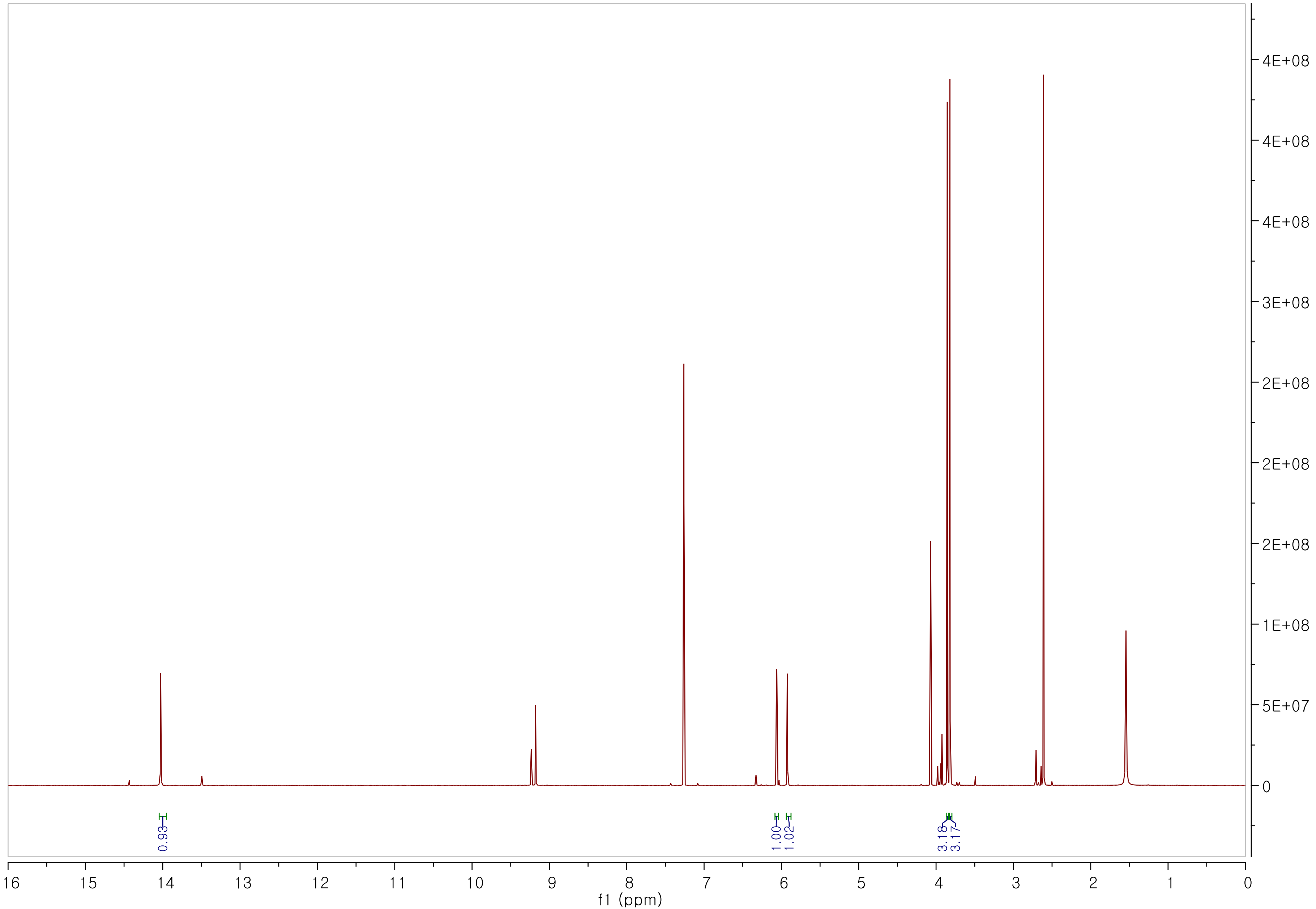

Supplement: Supplementary Materials — Table S1: the herbarium records of Zanthoxylum species. Figure S1: the 1H NMR spectrum of xanthoxylin mixed with the internal standard, methyl 3,5-dinitrobenzoate. [file 3830258.f1.pdf]
